# The impact of Ethiopian community-based health extension program on diarrheal diseases among under-five children and factors associated with diarrheal diseases in the rural community of Kalu district, Northeast Ethiopia: a cross-sectional study

**DOI:** 10.1186/s12913-022-07565-7

**Published:** 2022-02-09

**Authors:** Ahmed Tadesse, Fasil Walelign Fentaye, Asnakew Molla Mekonen, Toyeb Yasine

**Affiliations:** Department of Health Systems Management, School of Public Health, College of Medicine and Health Sciences, Wollo University, Dessie, Amhara Ethiopia

**Keywords:** Health extension program, Impact, Diarrheal disease, Rural community, Kalu district, Ethiopia

## Abstract

**Background:**

The health extension program is a community-based health care delivery program with eighteen defined packages. The main aim of the health extension program is to help to reduce child mortality. So, the aim of this study is to assess the impact of a health extension program on diarrheal disease under-five children in the rural community of Kalu district, Northeast Ethiopia, 2021.

**Methods:**

A community-based cross-sectional study design was conducted from March to April/2021. A Multi-stage sampling technique was used to get a total sample size of 556 (182 model households and 374 non-model households) with a response rate of 92.22%. Binary logistic regression analysis was done, and *P*-value < 0.05 was considered statistically significant. Propensity score matching analysis was used to determine the contribution of health extension program “model households” on diarrhea diseases among under-five children. The average treatment effect on the treated was calculated to compare the means of outcomes across model and non-model households.

**Results:**

Health extension program (HEP) model household contributed a 17.7% (t = -5.02) decrease in children’s diarrheal diseases among under-five children compared with HEP non-model households. Mothers from non-model households were 2.19 times more likely to develop under-five children diarrheal diseases AOR (Adjusted Odds Ratio): 2.19, 95% CI: 1.34–3.57 than mothers from model households. Households who got no frequent home visits were 3.28 times more likely to develop under-five diarrheal diseases AOR (Adjusted Odds Ratio): 3.28, 95% CI: 1.40–7.68.

**Conclusion:**

When the health extension program is implemented fully (model household), the prevalence of under-five diarrheal disease in the rural community could decrease. The need to develop supportive strategies for the sustainability of model households and encouraging households to be model households is very important.

**Supplementary Information:**

The online version contains supplementary material available at 10.1186/s12913-022-07565-7.

## Background

The Alma-Ata Declaration of 1978 recognized primary health care (PHC) as the most approach to the attainment of the objective of “Health for All”. The PHC approaches the most health issues in the community through the arrangement of basic health services. The execution of the PHC approach depends on healthcare workers including health extension workers (HEW) [1, 2]. Endeavors by the government to grow the PHC framework and stress preventive, promotional, and basic curative health services brought in encouraging changes in health coverage and utilization [3–5].

The health extension program is a community-based health care delivery program with eighteen defined packages [6]. The program was officially rolled out by the Ethiopian Federal Ministry of Health in 2003 and became implemented after the graduation of 7,136 health extension workers-trained to work mainly in disease prevention and health promotion in rural villages [7]. The program has four health subprograms; disease prevention, family Health; environmental hygiene and sanitation; and health education. The health extension program helps to reduce child mortality and maternal mortality and food and water hygiene, health housing management, solid and liquid waste management, personal hygiene, child health and immunization are the packages that influence under-five diarrheal diseases (see S1.table) [8, 9].

Diarrheal infection is transmitted through contaminated food or drinking water or from person-person as a result of poor hygiene. Globally, 9% of under-five deaths are due to diarrhea and the burden of diarrhea disease remains high [10]. Some ways to reduce the risk of diarrhea are safe drinking water, the use of improved sanitation and hand washing with soap [11].

About 7.2 million children died under the age of 5 years globally and of them 1.3 million children died due to preventable diarrhea and was the second leading cause in 2012 [9]. The majority of these deaths occur in India, Nigeria, Afghanistan, Pakistan, and Ethiopia [12]. Studies have suggested that diarrhea was even more likely in children with HIV, and the leading cause of death among HIV-infected infants. Persistent diarrhea adds to mortality by causing malnutrition and wasting that weaken the children's immunity [10, 13].

Even if improvement were made in reducing childhood mortality from 88 under-five deaths per 1,000 live births in 2011 [14] to 67 under-five deaths per 1,000 live births in 2016 in Ethiopia, children in the country still suffer from diarrhea [15]. In Ethiopia, about 90% of diarrhea disease occurs due to poor sanitation, lack of access to clean water supply, and inadequate personal hygiene which can be easily improved by health promotion and education [16]. Health promotion and education is an important aspect of primary health care [17].

The studies done in different areas of the globe showed that risk factors related to childhood diarrhea diseases were the child’s sex, child’s age, husband's education level, mother’s work status, mother’s marital status, breastfeeding status, and socio-economic status of the household [18–23]. But a study in Iran showed that the sex of a child is not associated with under-five diarrheal diseases [24]. Additional studies conducted in three South Asian countries, and Bolivia showed that a higher level of formal educational status of the caregiver or mother was protective against childhood diarrhea disease [21, 23, 25, 26]. Other studies were done in Latin American countries, India, Indonesia, Iran, and Tanzania, and they showed that the age of the mother or caregiver, the age of children, and the age of children had a significant effect on childhood diarrheal disease [19–22, 24, 26]. Studies conducted in Ethiopia and West African countries showed that the low level of husband education, private workers of mothers’ and farmer fathers’ occupation, large family size, older children having more risk for diarrheal disease [18, 27–32].

Studies done in Bangladesh and Ethiopia revealed that hand washing at recommended times, food preparation, hand washing, and family fetch water storage containers affected under-five children's diarrheal diseases [27, 33, 34]. But other studies showed that there was no significant association between hand washing with or without soap before feeding a child or after cleaning a child’s anus who has defecated and washing at a critical time with child diarrhea [33, 35, 36]. Also, another study in Ethiopia revealed that children whose mother didn’t practice hand washing at a critical time and families did not treat drinking water had more likely to concede childhood diarrhea [11, 18, 35]. Different studies done in Africa revealed that the child toilet, the child’s water storage container, water sources, the type of toilet, and lack of latrine ownership had a significant contribution to childhood diarrhea [31, 32, 37, 38]. Studies were done on different community health programs in Nepal (training and engaging community health volunteers) [39], Kenya (community unit performance), South Africa (community health worker home visits), Southern Asian countries (community health workers), Ethiopia (health extension program), revealed diarrheal diseases less likely occurred [11, 40–43].

The national scale-up of integrated community case management (ICCM) in 2010–2012 provided a needed boost to the HEP by introducing a package of high-quality basic curative interventions meeting the demand of the communities. According to the national ICCM guidelines, a health extension worker (HEW) assesses and classifies newborn infections and treats them. If a severe infection is diagnosed, refer after the first pre-referral management [44]. Oral rehydration salt (ORS) and zinc supplements as management of under-five diarrheal diseases recommended by the united nation international children’s fund (UNICEF) and the world health organization (WHO) since 2004 [45]. The health extension program enabled Ethiopia to achieve significant improvements in maternal and child health, communicable diseases, hygiene and sanitation, knowledge, and health care seeking [7].

The HEP is one means of implementing the sustainable development goal (SDG) by bringing main maternal, neonatal, and child health interventions to the community [46]. A model household is a household graduated by the health extension program after fulfilling health extension packages, but a non-model household is a household not graduated. Although HEP was implemented in 2005, the impact of HEP on child diarrhea was not investigated in this study area as far as my knowledge is concerned. So, the investigator wants to fill these gaps. The objective of this study is to assess the impact of HEP on diarrheal disease among under-five children and to identify factors associated with diarrheal disease in the rural community of Kalu district, Northeast Ethiopia.

## Methods

A community-based cross-sectional study was carried out from March 20 to April 20/ 2021. The study was conducted in Kalu district, Amhara regional state, Northeast Ethiopia. The 2021 district population projection was 238,162 of which 51% were males. Additionally, the total numbers of households in the district are 55,323. In the Kalu district, there are nine health centers, 33 health posts and 73 health extension workers. About 70% of households are models and the rest are non-model households. The district has 35 rural kebeles. Kebele is the smallest administrative unit in Ethiopia which comprises 5000 people.

The study population for model households and non-model households was all households with under-five children from randomly selected kebeles in the rural community of Kalu district, Northeast Ethiopia during the data collection period. Households who had permanent residence in the area for at least 12 months and households classified as model households by data collectors by the standard checklist on the data collection period were included in the study. For non-model households’ families who had permanent residence in the area for at least 12 months and did not fulfill or resist implementing the health extension program packages were included. But households with critically ill household mothers or caregivers during the study period were excluded from the study.

The required sample size was calculated by using Epi-Info version 7.2 through assumption of 95% confidence level, 5% margin of error (d), 80% power,1:2 ratio for model to non-model household, AOR (Adjusted Odds Ratio) = 2.25 and the percentage of outcome unexposed to pit or flush toilet (*p* = 57.2) from a previous study [11], design effect two and 10% non-response rate which yielded 603 participants (201 model household and 401 non-model households).

A multi-stage sampling procedure was employed to select study participants. First seven Kebeles from the thirty-five Kebeles were selected randomly using a lottery method and households who had under five children were identified in each kebele [47]. The kebeles were Adame (1818 households), Beke (1588 households), Qedida (870 households), Agamsa (1465 households), Jejeba (860 households), Mekanity (2394 households), and Ardibo (1656 households). Then proportionally the study participants allocated for each kebele. By using 1:2 ratio (model to non-model household) the allocated proportions in each kebele divided. Data found from each kebele (family folder) was used to identify model and non-model households. Then by using a sampling frame from the family folder, a simple random sampling method was used to select model and non-model household.

Data was collected from model and non-model households using an interviewer administered structured questionnaire. The questionnaire was adapted from other different studies [11, 28–30, 48, 49]. The questionnaire was prepared primarily in English, then it was translated to the local language Amharic and back-translated to English by language experts. Data collection tools mainly measure; socio-demographic characteristics, environmental-related factors, behavioral-related factors, HEP related factors, and clinical-related factors of the child.

### Operational definitions

The dependent variable was the existence of under-five children with diarrheal disease, and the treatment variable is health extension program. The independent variables were; socio demographic characteristics (residence, wealth index, parental education, maternal occupation, maternal age, child age, house hold size, number of children < 5 year), environmental characteristics (distance to water source, availability of latrine, availability of hand washing facility, daily per capita water consumption, refuse disposal), behavioral factors (method of water storage, hand washing practice, feeding practice, duration of breast-feeding, breast feeding status, time of introducing supplementary feeding, home based water treatment), and health extension program related factors (model household, home to home visit, health post visit).

Diarrheal disease is usually loose and watery stools, and at least three times in a 24-h period and the mother or care giver says diarrhea in the last two weeks [50] before data collection. The model household is a household graduated from the health extension program after fulfilling health extension packages and those who have a graduation certificate and continuity of package use or greater than or equal to eighty-five point from hundred by standard checklist assessment. But a non-model household is a household which had less than eighty-five points from hundred by standard checklist of model household measurement during data collection time. The cutoff point 85 is used as standard in the Amhara regional health bureau (see S2.table) [8]. Hand washing at critical time is if a mother/caregiver practiced all hand washings before food preparation, before child feeding, after child cleaning and after latrine visiting was considered “all practiced” unless considered as “partially practiced”. Proper refuse disposal is a way of waste disposal which includes burning, buried in a pit or store in a container, compost, and disposed in a designed site, whereas disposing in an open field was considered improper refuse disposal.

### Data quality control

The training was given for data collectors and supervisors for two days on information about the research objective, eligible study participants, data collection tools and procedures, and interview methods. Additionally, day-to-day supervision during the whole period of data collection was provided by the principal investigator. The data collection instruments were pre-tested on 50 households in Kutaber district, Northeast Ethiopia two weeks before the actual data collection period and revised accordingly.

The filled questionnaire was checked for completeness and accuracy by both data collectors and supervisors before they returned from the field. Typographic errors were manually edited, but incomplete questionnaires were considered non-response rates. Every questionnaire was checked by the principal investigator every day after data collection before data entry.

### Data analysis procedures and management

Data were entered to Epi-data version 3.1 and exported to Statistical Package for Social Science (SPSS) version 26 for cleaning, coding and analysis. Data consistency and missing values were checked before analysis. Descriptive statistics were computed for the prevalence of the model and non-model households. To identify factors independently associated with diarrhea, logistic regression models were fitted. The model fitness was checked by using Hosmer and Lemeshow goodness of test. Multi-collinearity was checked by using the variance inflation factor (VIF) or tolerance test. Variables *p*-value < 0.25 in bi-variable analysis was considered for binary logistic analysis. Data was also interpreted by using Odds ratio with 95% confidence level. 95% CI for the proportion was calculated by bootstrapping (100 replication). The principal component analysis was used to compute the wealth index.

Propensity score matching (PSM) is a quasi-experimental method in which the researcher uses statistical techniques to construct an artificial control group by matching each treated unit (in this case HEP model households) with a non-treated unit (in this case HEP non-model households) of similar characteristics. Using these matches, the researcher can estimate the impact of an intervention (HEP on the diarrheal disease). Kernel matching was used to match the HEP model and non-model households based on propensity scores. Kernel matching applied because it uses all data which maximizes information gain.

Propensity score matching analysis was performed by using STATA version 14.1 to determine the contribution of health extension program “model households” on under five years child diarrhea diseases. The standard error was computed through bootstrapping with 100 replications to adjust for the additional sources of variability introduced by the estimation of the propensity score and the matching process itself. The average treatment effect on the HEP model household (ATT) was calculated by averaging the difference between the under-five diarrheal disease of the model households and that of the non-model households after matching using propensity score. A t-test between the outcomes for the model and non-model households and 95% CI were computed. A wealth index, child sex, age of mother, occupation of mother, hand washing practice at the critical time, birth order of the child, number of people in the house, water container, and time to fetch water, main water sources, and age of the child were the variables used to construct it.

In general, a program (in this case HEP) evaluation using a propensity score matching requires a series of steps. First, fitted probit model using pre-intervention/pre-exposure covariates to estimate the propensity that a household is included in the treatment (T = 1) or not (T = 0). Second, and upon estimating the propensity scores, a relevant matching estimator is called for to match the treatment observations with comparable comparison observations using the propensity scores (in this research using Kernel matching). An important precursor to ensure the quality of matches is to impose what is known as ‘the common support condition’ in which 0 < *P* (T = 1/Z) < 1 is satisfied [51].

## Results

### Socioeconomic and demographic related characteristics

A total of 603 (201 models and 402 non-models) households that had at least one under-five child was planned to participate in the study. Out of these 556 (182 models and 374 non-models) were participated in the study, which makes a response rate of 92.2%. The mean age of the respondents (mother or caregiver) was 32.14 ± 6.23 years of age. Out of the participants, 536 (96.4%) were married, 182 (32.7%) were illiterate, 519 (93.3%) were farmers, 554 (99.9) were Muslims and 181 (32.6%) were illiterate fathers. The mean family size of the households was 5.36 ± 1.9 (Table 1).

**Table 1:** Socio-economic and demographic characteristics of households in Kalu district rural community, Northeast Ethiopia, 2021(*n* = 556)

Variable	Frequency	Percent
Age of mother or care giver		
24 and below	42	7.60
25- 35 years	328	59.00
Above 35 years	186	33.30
Marital status of respondent		
Single	6	1.10
Marriage	536	96.40
Widowed	7	1.30
Divorced	7	1.30
Educational status of the mother		
Illiterate	182	32.70
Read and write	46	8.30
1–6 grade	175	31.80
7–8 grade	77	13.80
9–12 grade	67	12.10
Diploma and above	9	1.60
Parent’s religion		
Muslim	554	99.60
Orthodox	2	0.40
Educational status of the father		
Illiterate	181	32.60
Read and write	60	10.80
1–6 grade	138	24.80
7–8 grade	86	15.50
9–12 grade	79	4.20
Diploma and above	12	2.20
Occupation of mother		
Farmer	519	93.30
Other	37	6.70
Family size of the household		
5 or less	300	54.00
6 and above	256	46.00
Wealth index		
Poor	295	53.10
Medium	92	16.5.00
Rich	169	30.40
Relationship with the child		
Mother	541	97.30
Care giver	15	2.70

### The health extension program status of the households’ characteristics

A total of 554 (99.6) heard about HEP of which 364 (65.5%) were heard information from health extension workers. Among them, 374 (63.7%) of the household were non-model, and 300 (54%) did not accurately mention the health extension packages. A total of 542 study participants (97.5%) had home visits by HEWs, and 283 (50.9%) had frequent visits (at least one visit every 4 weeks). A total of 556 (100%) of the participants visit the health post-visit (Table 2).

**Table 2:** HEP related characteristics of the households in Kalu district rural community, Northeast Ethiopia, 2021(*n* = 556)

Variable	Frequency	Percent
Heard about HEP		
Yes	554	99.60
No	2	0.40
Sources of information (554)		
HAD	99	17.80
WDA	88	15.80
HEW	364	65.50
Mass media	3	0.50
HEP status of the household		
Model household	182	32.70
Non model household	374	67.30
Number of HEP packages		
Accurately mentioned	300	53.96
Not accurately mentioned	240	43.16
I don’t know	16	2.88
Home to home visit by health extension worker		
Yes	529	95.14
No	27	4.86
Frequency of home visit		
No visit	27	4.90
Less frequent visit	246	44.20
Frequent visit	283	50.90
Health post visit by the community		
Yes	556	100.00
No	0	0.00

*HAD* Health Development Army, *HEW* Health Extension Worker, *WDA* Women Development Army

### Environmental characteristics of the participants

A total of 510 (97.1%) and 155 (30.39%) of the households had a latrine and hand washing facility respectively. The majority of the latrine facilities of the households were private, 502 (98.43%) but 33 (6.47%) were not improved. Out of the total households, 244 (43.88%) households take 15–30 min to fetch water. Only 281 (50.5%) of the households do not treat their drinking water at home (Table 3).

**Table 3:** Environmental characteristics of the households in Kalu district rural community, Northeast Ethiopia 2021(*n* = 556)

Variable	Frequency	Percent
Latrine availability		
Yes	510	91.73
No	46	8.27
Ownership of latrine		
Private	502	98.43
Common	8	1.57
Types of latrines		
Improved	477	93.53
Not improved	33	6.47
Latrine location from water sources		
Uphill	163	31.96
Same level	169	33.14
Downward	178	34.90
Hand washing facility near to latrine		
Yes	155	30.39
No	355	69.60
Time to fetch water (minutes)		
Less than 15	182	32.70
15–30	244	43.90
More than 30	130	23.40
Water container		
Cover	555	99.80
No cover	1	0.20
Water consumption (L/p/d		
≤ 7	164	29.50
> 7	392	70.50
Water treatment at home		
Yes	275	49.50
No	281	50.50

### Behavioral related characteristics of the participants

A total of 65.6% of households perform improper disposal methods. Besides, 28.04% of the households properly utilized the latrine, 69.96% of the households properly practiced children’s stool disposal, and 37.77% of respondents practiced hand washing at critical times. Soap utilization for hand washing was practiced at 52.16% of respondents (Table 4).

**Table 4:** Behavioral characteristics of households in Kalu district rural community, Northeast Ethiopia, 2021(*n* = 556)

Variable	Frequency	Percent
Refuse disposal method		
Proper	191	34.40
Improper	365	65.60
Proper latrine utilization		
Proper	143	28.04
Improper	367	71.96
Children stool disposal methods		
Proper	389	69.96
Improper	167	30.04
Hand washing at critical time		
All practiced	210	37.77
Partial practiced	346	47.84
Soap utilization for hand washing		
Yes	290	52.16
No	266	47.84

### Demographic and health characteristics of the index child

Of all the under-five children (53.4%) were males, 220 (39.6%) and (98.56%) received the Rota virus vaccine (Table 5).

**Table 5:** Demographic and health characteristics of the index child in Kalu district rural community, Northeast Ethiopia, 2021 (*n* = 556)

Variable	Frequency	Percent
Age		
< 6 months	87	15.60
6–12 month	125	22.50
12–24 month	163	29.30
> 24	181	32.60
Sex		
Male	297	50.40
Female	259	46.60
Birth order		
1	118	21.20
2–3	220	39.60
4–5	186	33.50
> 6	32	5.80
Rota vaccination of the child		
Yes	548	98.60
No	8	1.40
Occurrence of diarrhea in last 2 weeks		
Yes	149	26.80
No	407	73.20

### Diarrheal disease prevalence

The prevalence of diarrhea in the previous two weeks before data collection of the survey was 26.8% (149) % (95% CI: 23.2–30.6). From this figure, 14.28% were from model houses and 32.89% were from non-model households.

### Factors associated with under-five child diarrheal diseases

HEP status of the household, frequency of home visits by HEW, and hand washing at the critical time were statistically associated with under-five children diarrheal disease. The likelihood of developing under-five child diarrheal disease from HEP non-model households was 2.19 times AOR (Adjusted Odds Ratio): 2.19, 95%CI: 1.34–3.57 more likely as compared to HEP model households. Among the study participants, households who did not get frequent home visits were 3.28 times AOR (Adjusted Odds Ratio): 3.28,95%CI: 1.40–7.68 to develop under-five child diarrheal diseases as compared to households who had gotten frequent home visits (at least one visit within a month by a health extension worker). Children whose mothers did partial hand washing at the critical times were 2.85 times AOR (Adjusted Odds Ratio): 2.85, 95% CI: 1.78–4.56 more likely to develop under-five diarrheal diseases than children whose mothers practiced hand washing at critical times (Table 6).

**Table 6:** Factors associated with under five children diarrheal disease in Kalu district rural community, Northeast Ethiopia, 2021(*n* = 556)

Variable	Diarrheal disease		AOR (95% CI)
	Yes	No	
Age of mother or care giver			
24 and below years	17	25	1
25 -35 year	83	245	0.62(0.34–1.14)
Above 35 years	49	137	0.74 (0.32–1.74)
Wealth index			
Poor	86	209	1
Medium	19	73	0.62(0.30–1.35)
Rich	44	125	0.86(0.54–1.40)
HEP status of the household			
Model household	26	156	1
Non model household	123	251	2.17(1.33–3.55)*
Frequency of home visit			
Frequent visit	69	214	1
Less frequent	66	180	1.04(0.69–1.56)
No visit	14	13	3.24 (1.37–7.58)*
Hand washing at critical time			
All practiced	29	181	1
Partial practiced	120	226	2.92(1.82–4.66)**
Time to fetch water			
Less than 15 min	42	143	1
15 to 30 min	75	166	1.4 (0.86–2.33)
More than 30 min	32	98	1.1(0.61–1.99)
Child age in month			
Up to six months	20	67	1
From six to 12 month	26	99	0.96 (0.46–1.99)
From 12 to 24 month	44	119	1.20 (0.6–2.53)
Above 24 months	59	122	1.56 (0.80–3.03)
Birth order of child			
1	39	79	1
2–3	56	164	0.73 (0.44–1.23)
4–5	47	139	0.6 (0.35–1.02)
6 and above	7	25	0.48(0.2–1.27)
Occupation of mother			
Farmer	142	377	1
Other	7	30	0.6(0.24–1.06)
Sex of child			
Male	71	226	1
Female	78	181	1.42 (0.96–2.11)
Number of people in the house			
≤ 5	81	188	1
> 5	68	68	1.69 (0.89–3.34)

*AOR* Adjusted Odds Ratio, *CI* Confidence Interval,*P-value < 0.05 **p-value < 0.0001

### Impact health extension program on under-five diarrheal diseases

The study showed that the average treatment effect on treated model households (ATT) was found to be -0.177 points (t = -5.02) 95% CI: -0.25 -0.11) for under five-year child diarrheal diseases. This indicated that HEP contributed 17.7% decrease in diarrheal diseases under five children diarrheal diseases compared with non-HEP implementing households (Table 7).

**Table 7:** ATT of HEP “model households” on under five diarrheal diseases by the community, Kalu district rural community, Northeast Ethiopia, 2021(number of replications = 100)

Dependent variable	Model households	Non-model households	ATT	SE	*t*	95% CI
Under five diarrheal diseases	182	362	-0.177	0.04	-5.02	-0.25 -0.11

Number of observations = 544

*ATT* Average Treatment effect on Treated, *CI* Confidence Interval, *HEP* Health Extension Program, *SE* Standard Error

**Fig. 1:**
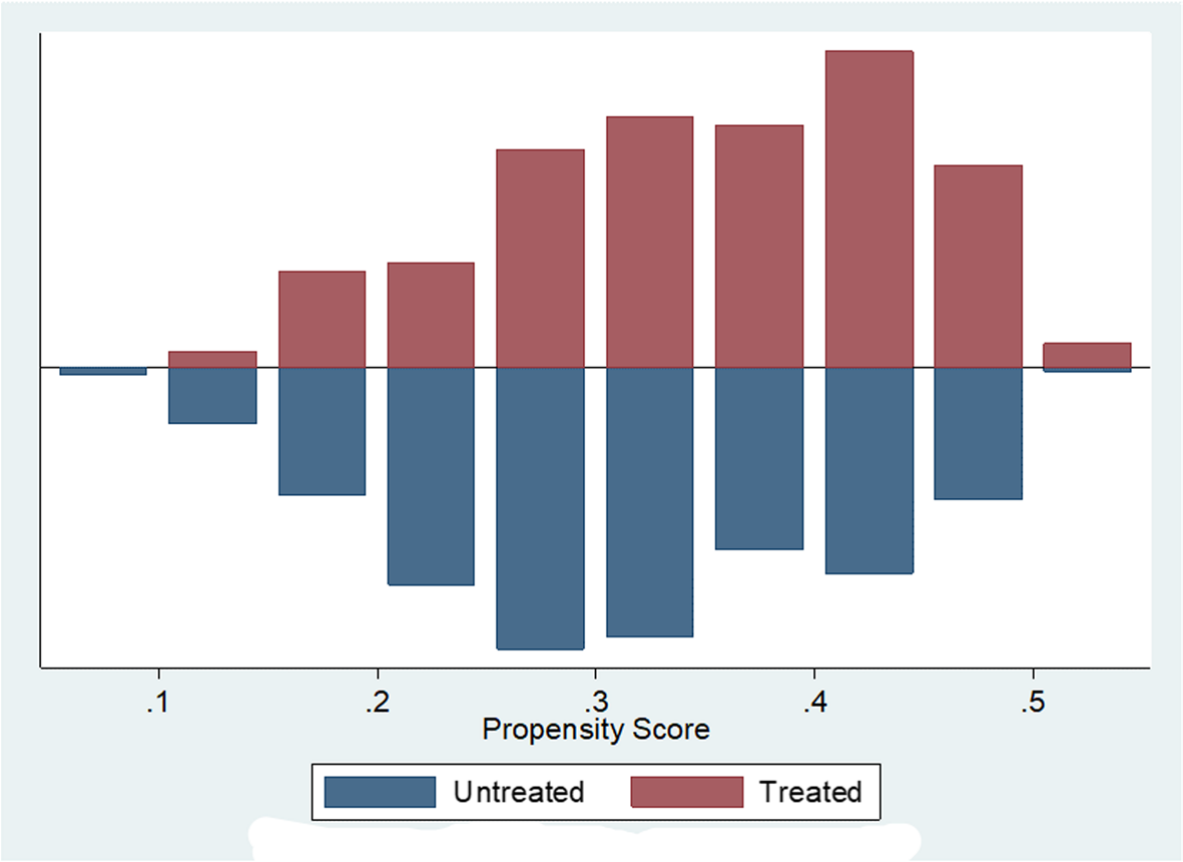
The common support region for model and non-model households

**Fig. 2:**
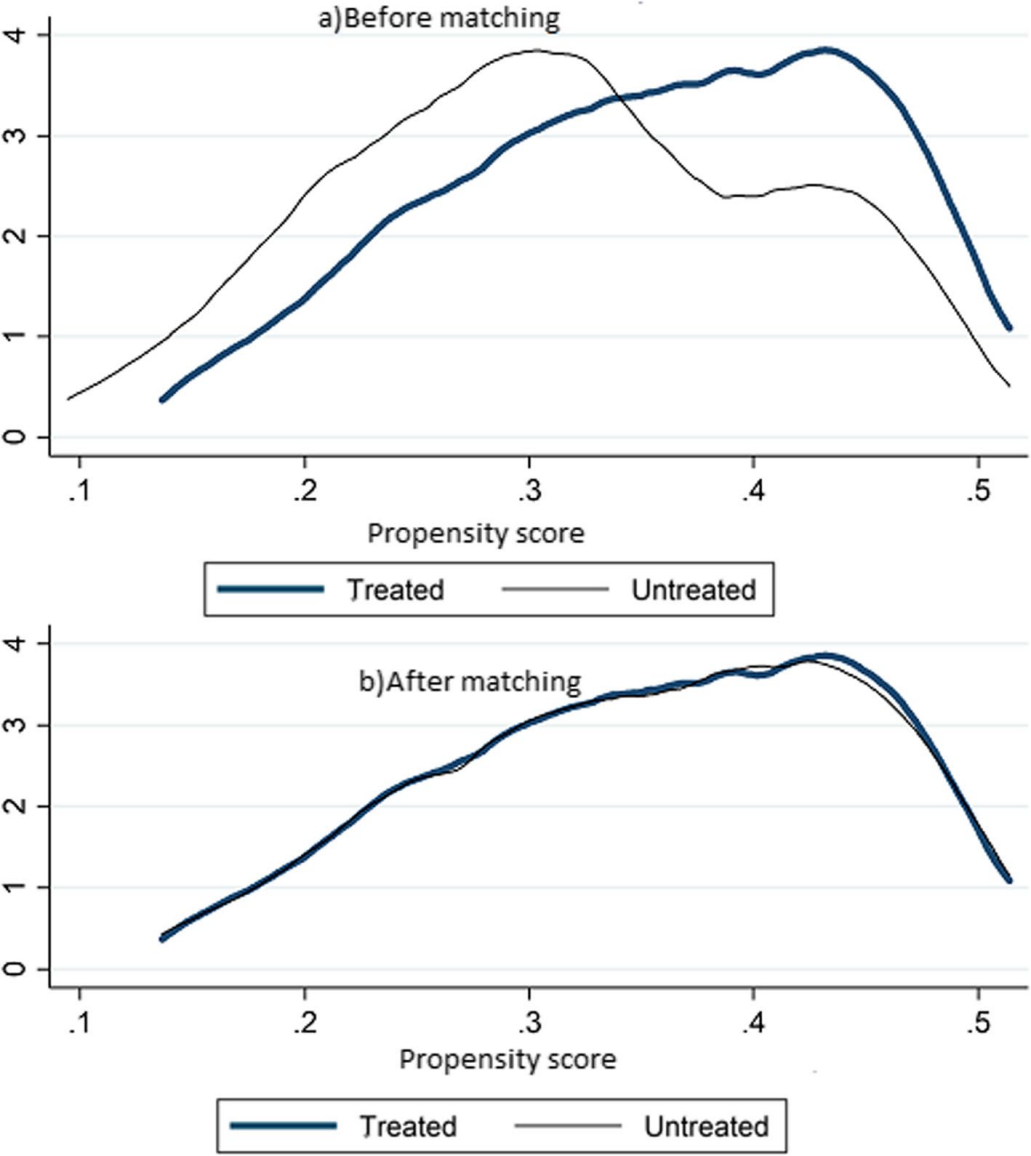
Propensity score distribution in the model and non-model households before and after matching

The common support region for model and non-model households (Fig. 1). Propensity score distribution in the model and non-model households before and after matching (Fig. 2).

## Discussion

The aim of this study was to assess the impact of a health extension program on diarrheal diseases among under-five children and to identify factors associated with under-five children diarrheal diseases in the rural community of Kalu district. Health extension program status, mothers or caregivers practice hand washing at a critical time and the frequency of home visits by health extension workers was statically associated with under-five children diarrheal diseases.

The prevalence of diarrheal disease among under-five children in this study was 26.8% (95% CI: 23.2–30.6). This finding is consistent with other studies done in different parts of Ethiopia which were, 27.2% Northern Ethiopia [52], 23.5% Southern Ethiopia [53], across regions of Ethiopia [16], 22.1% Western Ethiopia [28], and 23.1% Northeast Ethiopia [54] and 26.1% Cameron [53]. But the finding is higher with studies reported in different areas, 12% the national report [15], 14.7% Eastern Ethiopia [49], 13.6% South Ethiopia [55], 16.4% Debre Berhan city, and 13% Nigeria [29, 56]. The difference might be due to the difference in the socio-demographic characteristics, seasonal variation, sanitation, and basic environmental infrastructures of study households. Because the study on other areas showed that the highest average incidence rate was observed during the pre-rainy season (March to May) [57], and implemented community-led total sanitation and hygiene was a tool to reduce child diarrheal prevalence.

Being non-model households for the health extension program was more likely to develop under-five diarrheal diseases as compared to model households for the health extension program. This is consistent with a study done on the South Ethiopia rural community [11]. This is obvious the implementation of eighteen health extension packages have a positive effect on diarrheal diseases. Especially, food and water hygiene, solid and liquid waste management, personal hygiene, and immunization have a direct influence on diarrheal diseases.

The study also revealed that mothers who had not practiced hand washing at a critical time were more likely to develop diarrhea when compared to children whose mothers practiced hand washing at a critical time. This was consistent with the study finding where mothers or caregivers who lack hand washing practice and hand washing with water only contributed to under-five diarrheal disease [58]. Another study in eastern and northern Ethiopia showed that hand washing with soap complemented with hand hygiene promotion significantly decreased diarrheal episodes [59, 60]. This might be due to hand washing decreases the contamination of foods with microorganisms and which in turn prevent the occurrence of diarrhea and other hygiene related diseases.

Besides, households that did not have home visits by health extension workers (HEW) were more likely to develop under-five diarrheal diseases than those that had a frequent home visit by health extension workers. It is supported by studies in south Asia [43], South Africa [42], and other studies elsewhere [61–63]. This may be the health extension worker (community-based health workers) promoting personal and environmental hygiene during home visits and the best opportunity for behavioral change for the whole family and caregivers for their children. Thus, change in behavior affects the occurrence of diarrheal diseases in rural communities [64]. Another study done in Ethiopia showed that health extension workers' home visits improved the utilization of health services [65]. Particularly, increasing vaccine coverage, especially the Rota vaccine which decreased diarrheal diseases [66].

The study revealed that the health extension program had an interesting impact on under-five children's diarrheal diseases reduction. The propensity score matching analysis showed that being a model household decreased diarrheal diseases under-five children diarrheal disease by 17.7%. This is evidenced by government reports and different scientific researches [55, 61]. This may due to the implementation of eighteen health extension program packages such as hand washing facility near the latrine, latrine construction, and use, which may improve the personal hygiene of the mother or caregiver. Besides, mothers or caregivers in HEP model households participate in women development army conferences and health development army conferences, which is a tool to convince the mother for health extension packages.

There were certain limitations to the research. First, this study used a cross-sectional study design to assess the impact of HEP model households on diarrheal diseases among under-five children, so causal relationships between factors and under-five diarrheal diseases could not be determined, even though propensity score analysis could provide an option.

Additionally, to determine the impact of HEP (model households), the propensity score may not be as successful as randomized controlled trials. Because there was no baseline data on under-five diarrheal diseases before the HEP was implemented, we were unable to determine the HEP's actual contribution to under-five diarrheal diseases from the baseline, and propensity score ignores the effects of unobserved characteristics that may have an impact on the study's results. As a result, the outcomes of this study should be viewed in light of these factors.

## Conclusions

This study showed that the prevalence of the diarrheal disease among under-five children was high. Besides, it revealed that implementing a health extension program (being a model household) has a significant reduction of diarrheal disease among under-five children. Hand washing practice at a critical time, model household status, and frequency of home visits were significantly associated with under-five diarrheal diseases. Therefore, the health extension program is important to reach the United Nations (UN) target, by 2030, to end preventable deaths of newborns and children under 5 years of age, with all countries aiming to reduce neonatal mortality to at least as low as 12 per 1,000 live births and under-5 mortality to at least as low as 25 per 1,000 live births. Kalu District health office should scale up the number of model households from non-model households in order to reduce under-five diarrheal diseases. The health extension workers should frequently visit the households and educate the hand washing practice.

## Supplementary Information

**Additional file 1: Table S1. **The Ethiopian health extension program packages.

**Additional file 2: Table S2.** Model and non-model household assessment checklist.

## Data Availability

The datasets used and/or analyzed during the current study are available from the first author on reasonable request.

## Abbreviations

ATT: Average Treatment effect on the Treated
EDHS: Ethiopian Demographic health survey
HEP: Health Extension Program
HEW: Health Extension Worker
HIV/AIDS: Human Immune Virus/Acquired immune Deficiency Syndrome
HSDP: Health Sector Development Program
ICCM: Integrated Community Case Management
MDG: Millennium Development Goal
NGO: Non-Governmental Organization
ORS: Oral Rehydration Salt
PHC: Primary Health Care
UNICEF: United Nation International Children’s Emergency Fund
WHO: World Health Organization
SDG: Sustainable Development Goal
HDA: Health Development Army
WDA: Women Development Army
