# Supplementary material for: The impact of Ethiopian community-based health extension program on diarrheal diseases among under-five children and factors associated with diarrheal diseases in the rural community of Kalu district, Northeast Ethiopia: a cross-sectional study
Source: BMC Health Serv Res. 2022 Feb 9;22:168. doi: 10.1186/s12913-022-07565-7 (PMC8830013; doi:10.1186/s12913-022-07565-7)
Supplement: Supplementary file 1 — Additional file 1: Table S1. The Ethiopian health extension program packages. [file 12913_2022_7565_MOESM1_ESM.docx]

**S1 table: The Ethiopian health extension program packages**

| 1. **Hygiene and Environmental Sanitation** |
| --- |
| - 1. Food and water hygiene |
| - 1. Healthy housing construction and management |
| - 1. Solid and liquid waste management and disposal |
| - 1. Personal hygiene |
| - 1. Institutional hygiene |
| 1. **Family Health** |
| - 1. Maternal and neonatal health |
| - 1. Child health |
| - 1. Family planning and adolescent, youth and reproductive health service (AYRHS) |
| - 1. Immunization |
| - 1. Nutrition |
| 1. **Diseases and accidents prevention and control** |
| - 1. HIV/AIDS, and STI prevention and control |
| - 1. TB and leprosy prevention and control |
| - 1. Malaria prevention and control |
| - 1. First aid emergency measures |
| - 1. Non-communicable diseases prevention and control |
| - 1. Mental health |
| 3.7 NTD prevention and control |
| 1. **Health education and communication** |
| - 1. Health education and communication |

Source: Second generation health extension program revised, FMOH Ethiopia, 2019
