# Supplementary material for: The impact of Ethiopian community-based health extension program on diarrheal diseases among under-five children and factors associated with diarrheal diseases in the rural community of Kalu district, Northeast Ethiopia: a cross-sectional study
Source: BMC Health Serv Res. 2022 Feb 9;22:168. doi: 10.1186/s12913-022-07565-7 (PMC8830013; doi:10.1186/s12913-022-07565-7)
Supplement: Supplementary file 2 — Additional file 2: Table S2. Model and non-model household assessment checklist. [file 12913_2022_7565_MOESM2_ESM.docx]

| **S2.table Model and non-model household assessment checklist** | | | | | |
| --- | --- | --- | --- | --- | --- |
| S. No | Main functions | General  Weight | Parameters | Point | Remark |
| 1 | Regarding planning | 5 | She has a written plan, she is working on the plan | 5 |  |
|  |  |  | She has a written plan but has not put it into practice | 3 |  |
|  |  |  | She has no written plan | 0 |  |
| 2 | Her participation in the 1 to 5 and her health development team | 5 | If she is a regular participant | 5 | Confirmed by the combination |
|  |  |  | If you cut occasionally | 3 |  |
|  |  |  | If she is not involved | 0 |  |
| 3 | mother and child health services | 45 | Monthly Pregnancy Conference Participation (3) |  |  |
|  |  |  | If she is a regular participant | 3 |  |
|  |  |  | If she occasionally participates | 1 |  |
|  |  |  | If you do not participate | 0 |  |
|  |  |  | Prenatal Health Service (5) |  |  |
|  |  |  | From the 16th week onwards, she has been monitoring the pregnancy continuously according to the program | 5 |  |
|  |  |  | She stopped 3 times, | 3 |  |
|  |  |  | She stopped 2 times, | 2 |  |
|  |  |  | She only stopped once, | 1 |  |
|  |  |  | Unsupervised, | 0 |  |
|  |  |  | delivery Service (12) |  |  |
|  |  |  | She gave birth at a health center / hospital, | 12 |  |
|  |  |  | She gave birth at home | 0 |  |
|  |  |  | Postnatal Services ( 5) |  |  |
|  |  |  | Follow up by 48 hours after delivery | 5 |  |
|  |  |  | Follow up by 72 hours after delivery | 3 |  |
|  |  |  | If she / she did within a week of giving birth | 2 |  |
|  |  |  | If she follows up a week later | 1 |  |
|  |  |  | If she has no follow-up | 0 |  |
|  |  |  | Family Planning Service 5 |  |  |
|  |  |  | She has been a regular beneficiary of family planning services for up to 2 years and more | 5 |  |
|  |  |  | Vaccination 10 |  |  |
|  |  |  | She is vaccinating her child according to the immunization schedule | 10 |  |
|  |  |  | She started vaccination of her child but did not complete it within a year | 5 |  |
|  |  |  | She did not start vaccination of her child | 0 |  |
|  |  |  | Nutrition (5) |  |  |
|  |  |  | Exclusive breast feeding up to six week (if no contra indication) and /or start complementary feeding at six week and breast feeding up to 2 years  For less than 2 years of child weight and MUAC measurement every month | 5 |  |
|  |  |  | 2¼3 | 3 |  |
|  |  |  | 1¼3 | 1 |  |
|  |  |  | 0 | 0 |  |
| 4 | Hygiene and sanitation | 32 | Personal Hygiene 3 |  |  |
|  |  |  | If family members are kept clean | 3 |  |
|  |  |  | If there is a lack of hygiene | 0 |  |
|  |  |  | Setting up and using the toilet properly 12) |  |  |
|  |  |  | The toilet is protected and covered, If the floor is clean and She put hand-washing water and ash soap next to the toilet if she was using it properly and made others use it | 12 |  |
|  | Hygiene and sanitation |  | The toilet is protected and covered, If the floor is clean but not She put hand-washing water and ash or soap next to the toilet if she was using it properly and made others use it | 8 | Not office users 0 |
|  |  |  | The presence of a toilet and the use of it but the floor without protection and cover and also the floor is not clean | 4 |  |
|  |  |  | If all of the above was absent | 0 |  |
|  |  |  | Preparation and use of sewers(4) |  |  |
|  |  |  | (Compost or pit for composers and separately pit for decomposers  -seepage or pit for liquid waste disposal | 4 |  |
|  |  |  | ½ | 2 |  |
|  |  |  | 0 | 0 |  |
|  |  |  | Housing condition (10) |  |  |
|  |  |  | Separated house for animal and human  -house has window  shelf made of local material  -house and compound hygiene | 10 |  |
|  |  |  | 4 | 7 |  |
|  |  |  | 3 | 5 |  |
|  |  |  | 2 | 2 |  |
|  |  |  | 1 | 1 |  |
|  |  |  | 0 | 0 |  |
|  |  |  | drinking water (3) |  |  |
|  |  |  | -If you use safe drinking water or treated water-If you use a jerry can | 3 |  |
|  |  |  | From above mentioned one of fulfilled | 1.5 |  |
|  |  |  | From above mentioned one of fulfilled | 0 |  |
| 5 | Prevention and control of infectious diseases | 8 | Participate in community discussion in the health development army | 1 |  |
|  |  |  | Participate in community discussion in the health development army | 1 |  |
|  |  |  | The role of family members in preventing HIV / AIDS | 1 |  |
|  |  |  | Awareness of treatment when family members cough for two weeks or more | 2 |  |
|  |  |  | Use of ITN(3) |  |  |
|  |  |  | ITN used properly every day, keeping the ITN clean | 4 |  |
|  |  |  | If she is not a user | 0 |  |
| 6 |  | 5 | Use of CBHI (5) |  |  |
|  |  |  | CBHI user | 5 |  |
|  |  |  | CBHI not user | 0 |  |
| Total (%) | | 100 |  | 100 |  |
